# Identification of the key genes and microRNAs in adult acute myeloid leukemia with FLT3 mutation by bioinformatics analysis

**DOI:** 10.7150/ijms.46441

**Published:** 2020-05-18

**Authors:** Shuyi Chen, Yimin Chen, Zhiguo Zhu, Huo Tan, Jielun Lu, Pengfei Qin, Lihua Xu

**Affiliations:** 1 Department of Hematology, The First Affiliated Hospital of Guangzhou Medical University, Guangzhou, Guangdong 510000, China; 2 Department of Urology & Minimally Invasive Surgery center, The First Affiliated Hospital of Guangzhou Medical University, Guangdong Key Laboratory of Urology, Guangzhou Institute of Urology, Guangdong, China; 3 Department of Pediatrics, The First Affiliated Hospital of Guangzhou Medical University, Guangzhou, Guangdong 510000, China

**Keywords:** bioinformatics analysis, acute myeloid leukemia, FLT3 mutation, differentially expressed miRNAs and genes, miR-10a-3p

## Abstract

**Background**: Associated with poor prognosis, FMS-like tyrosine kinase 3 (FLT3) mutation appeared frequently in acute myeloid leukemia (AML). Herein, we aimed to identify the key genes and miRNAs involved in adult AML with FLT3 mutation and find possible therapeutic targets for improving treatment.

**Materials**: Gene and miRNA expression data and survival profiles were obtained from The Cancer Genome Atlas (TCGA) and Gene Expression Omnibus (GEO) database. EdgeR of R platform was applied to identify the differentially expressed genes and miRNAs (DEGs, DE-miRNAs). Gene ontology (GO) and the Kyoto Encyclopedia of Genes and Genomes (KEGG) enrichment analyses were performed by Metascape and DAVID. And protein-protein interaction network, miRNA-mRNA regulatory network and clustering modules analyses were performed by STRING database and Cytoscape software.

**Results**: Survival analysis showed FLT3 mutation led to adverse outcome in AML. 24 DE-miRNAs (6 upregulated, 18 downregulated) and 250 DEGs (54 upregulated, 196 downregulated) were identified. Five miRNAs had prognostic value and the results matched their expression levels (miR-1-3p, miR-10a-3p, miR-10a-5p, miR-133a-3p and miR-99b-5p). GO analysis showed DEGs were enriched in skeletal system development, blood vessel development, cartilage development, tissue morphogenesis, cartilage morphogenesis, cell morphogenesis involved in differentiation, response to growth factor, cell-substrate adhesion and so on. The KEGG analysis showed DEGs were enriched in PI3K-Akt signaling pathway, ECM-receptor interaction and focal adhesion. Seven genes (LAMC1, COL3A1, APOB, COL1A2, APP, SPP1 and FSTL1) were simultaneously identified by hub gene analysis and module analysis. SLC14A1, ARHGAP5 and PIK3CA, the target genes of miR-10a-3p, resulted in poor prognosis.

**Conclusion**: Our study successfully identified molecular markers, processes and pathways affected by FLT3 mutation in AML. Furthermore, miR-10a-3p, a novel oncogene, might involve in the development of FLT3 mutation adult AML by targeting SLC14A1, ARHGAP5 and PIK3CA.

## Introduction

Acute myeloid leukemia (AML) is a common hematological malignancy which the incidence is increasing 1. According to recent studies, a major reason why AML patients with low cure rate is drug resistance, usually manifested as relapse form remission 2. Understanding the mutation status of various genes in early diagnosis could improve the effect of initial treatment.

FMS-like tyrosine kinase 3 (FLT3), a type III receptor tyrosine kinase (RTK), is involved in multiple intracellular signaling pathways 3, 4. Activation of FLT3 plays an important role in hematopoietic cell survival, proliferation and differentiation 5, 6. However, FLT3 mutation is one of the most common genetic abnormalities in AML patients, accounting for about 30%-50%. And the most common type of mutation in the FLT3 gene is an internal tandem duplication (FLT3/ITD) 7. Associated with poor prognosis, mutated FLT3 is regarded as a promising therapeutic target for AML 8, 9. FLT3 inhibitors have approved for clinical use for mutant FLT3-positive AML in Japan and/or Europe and United states. But several resistance mechanisms of FLT3 inhibitors have been apparent in clinical studies 10. Further studies are still required to find novel biomarkers for improving therapeutic strategy in AML with FLT3 mutation 11. Chenglong Li et al. has investigated feature genes for predicting the FLT3/ITD mutation in pediatric and adult AML patients from the European Bioinformatics Institute (EBI) 12. But the study was a lack of some analyses, such as protein-protein interaction network, predicted microRNAs (miRNAs) and miRNA-mRNA regulated network.

Herein, to provide novel targets for treatment, we identified the key genes and miRNAs and better understood the main biological processes associated with FLT3 mutations in adult AML by bioinformatics analysis.

## Materials and Methods

### Data collection

In order to compare genes and miRNAs expression between FLT3 mutation and wild-type adult AML patients, gene and miRNA expression profiles and corresponding survival profiles were obtained from The Cancer Genome Atlas (TCGA) database (https://gdc-portal.nci.nih.gov/) 13. Simultaneously, the survival analysis data about FLT3 mutation and wild-type AML were provided by Bullinger L et al 14. The gene expression profile (GSE15434) for verification of target gene expression were downloaded from Gene Expression Omnibus (GEO) database (https://www.ncbi.nlm.nih.gov/geo/) 15. The basic information of the downloaded dataset was performed in Table 1.

### Identification of differentially expressed miRNAs (DE-miRNAs) and differentially expressed genes (DEGs)

The EdgeR was utilized to screen DE-miRNAs and DEGs according to user's guide 16, 17. P value<0.05 and |log_2_fold change (FC)|≥1 were set as the threshold values in DE-miRNAs, and P value<0.05 and |log_2_FC|≥1.5 were considered as cut-off criterion in DEGs.

### Enrichment analyses of DEGs

Gene ontology (GO) term analysis for the DEGs was performed using Metascape (http://metascape.org) 18, including biological process (BP), molecular function (MF) and cellular component (CC). KEGG pathways analysis for the DEGs was conducted by DAVID (https://david.ncifcrf.gov/tools.jsp) 19. P value<0.05 was considered statistically significant.

### Protein-protein interaction (PPI) network, miRNA-mRNA regulatory network, hub genes and module analysis

To analyze the connection among proteins, DEGs were uploaded to the Search Tool for the Retrieval of Interacting Genes (STRING, https://string-db.org/), which is a database that provides information on direct (physical) and indirect (functional) associations between different proteins 20. This database is designed to provide important assessments and integration of functional partnerships and interactions that occur between proteins. The PPI network of DEGs and the regulatory network of miRNA-mRNA were visualized by Cytoscape software 21. The cytoHubba plugin and Molecular Complex Detection (MCODE) plugin in Cytoscape were used to identify hub genes and screen modules of the PPI network 22, 23. All parameters of the plugin were left at their default values. GO and KEGG enrichment of hub genes and genes in modules were also analyzed by Metascape.

### Evaluation of the prognostic value of DE-miRNAs and target genes of miR-10a-3p

Survival curves were drawn by the Kaplan-Meier method. The hazard ratio (HR) with 95% confidence intervals (CIs) and log rank P value were analyzed by the Cox proportional hazards regression model to compare the overall survival (OS) of AML with different miRNA or mRNA expression.

### Prediction of target genes of DE-miRNAs

Target Scan 24, miRDB 25, miRPathDB 26 and miRWalk 27, target prediction databases, were both used to identify the target genes of DE-miRNAs. Moreover, the genes, predicted by the intersection of these four databases, were selected for further research.

### Statistical analysis

All the statistical analyses were conduct with SPSS version 20.0 and GraphPad Prism version 8.0. P value<0.05 was indicated as statistical significant.

## Results

### Procedure of bioinformatics analysis

**Figure 1:**
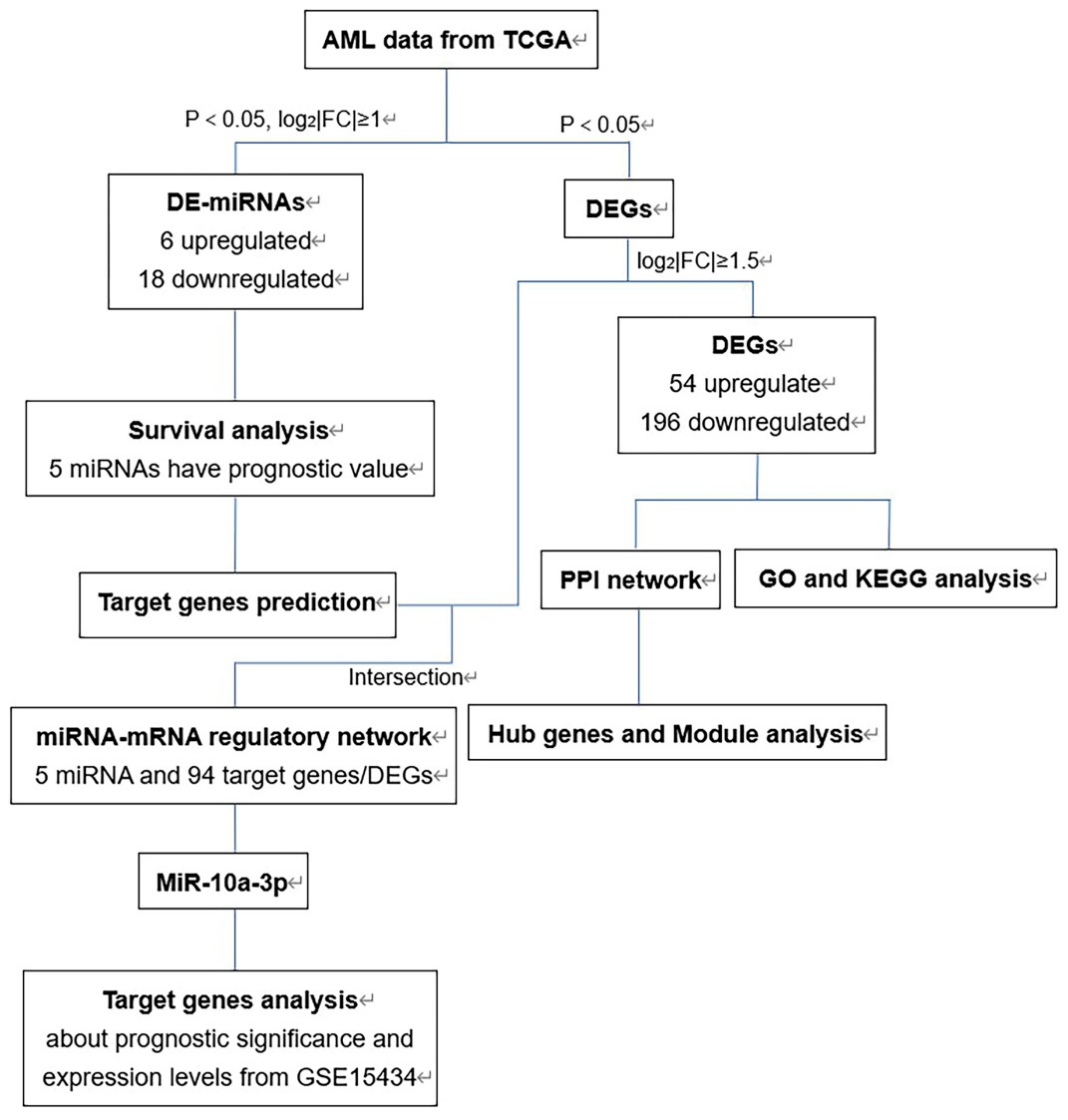
** The flow chart of bioinformatics analysis.** Abbreviations: AML, acute myeloid leukemia; TCGA, The Cancer Genome Atlas; DE-miRNAs, differentially expressed miRNAs; DEGs, differentially expressed genes; PPI: protein-protein interaction; GO: Gene ontology; KEGG: Kyoto Encyclopedia of Genes and Genomes.

The flow chart of bioinformatics analysis was shown in Figure 1. Firstly, we identified DE-miRNAs and DEGs of AML with FLT3 mutations from TCGA database and evaluated the prognostic significance of DE-miRNAs to select valuable DE-miRNAs. Secondly, DEGs were conducted to perform GO and KEGG enrichment analyses. Then we constructed PPI network of DEGs and performed hub genes and module analysis. Moreover, the valuable DE-miRNAs and their predicted target genes were together to build the miRNA-mRNA regulatory network. Finally, miR-10a-3p and its target genes were chosen to further analyses.

### Identification of DE-miRNAs and DEGs

**Figure 2:**
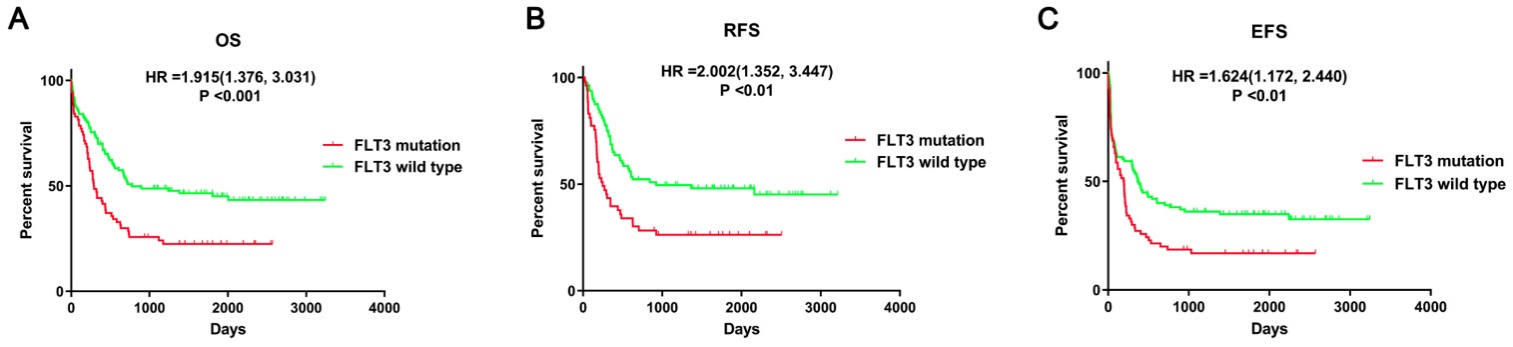
** Survival analysis between FLT3 mutant and wild-type AML.** Survival analysis was performed using data from Bullinger L et al. Abbreviations: OS: overall Survival; RFS: relapse-free survival; EFS: event-free survival.

First, the prognostic impact of FLT3 mutation in AML patients was evaluated using data from the article of Bullinger L et al. The survival analyses suggested that mutant FLT3 was significantly associated with poor overall survival (OS, P<0.001), relapse-free survival (RFS, P<0.01), event-free survival (EFS, P<0.01) in AML patients (Figure 2). Therefore, it is important to identify crucial miRNA and mRNA and understand the relevant processes and pathways affected by FLT3 mutation.

**Figure 3:**
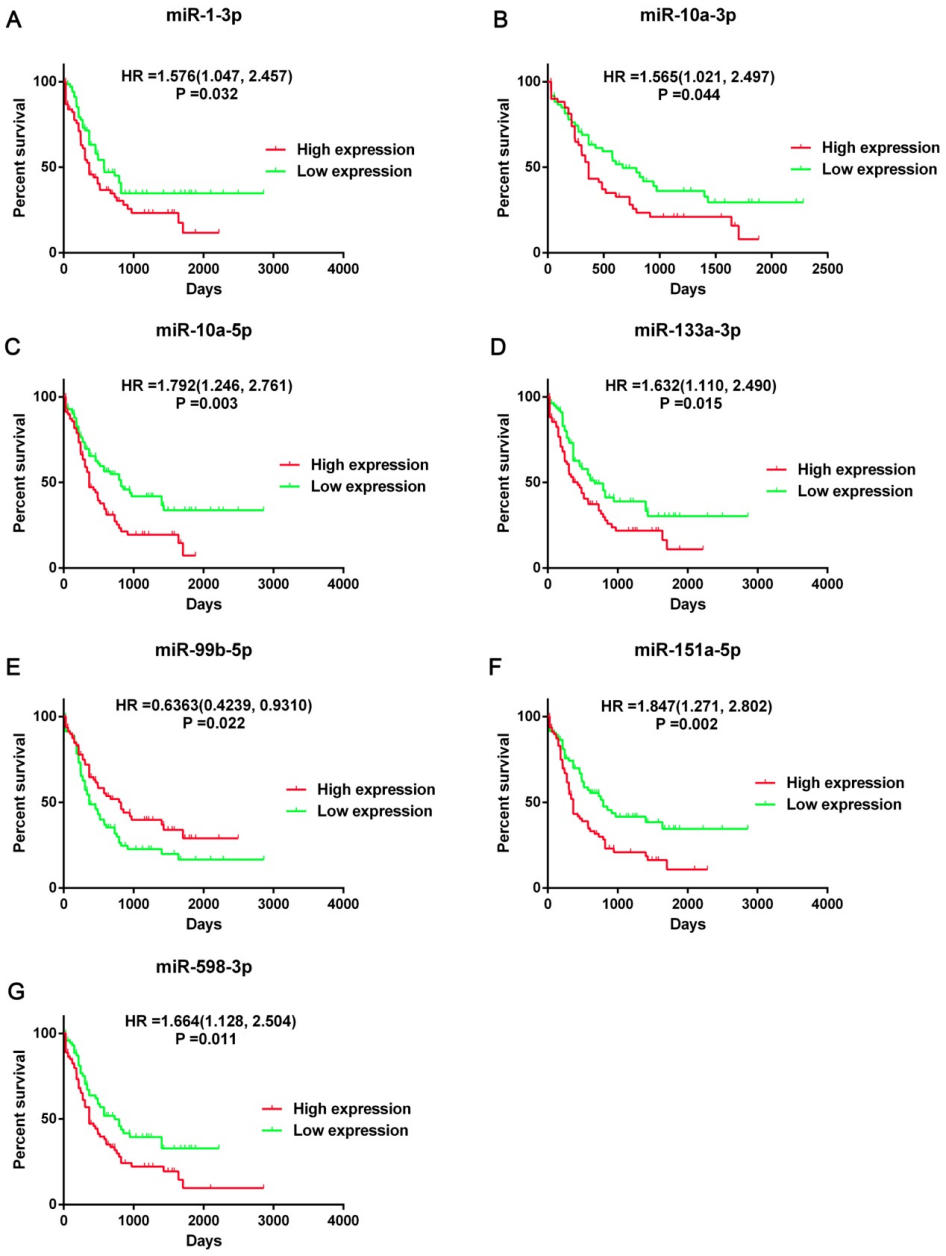
** Prognostic significance of seven DE-miRNAs.** Kaplan-Meier survival curves of overall survival (OS) in AML was performed using TCGA dataset. The top 50% miRNA expression was defined as high expression group and the rest was defined as low expression group. The hazard ratio (HR) with 95% confidence intervals (CIs) were analyzed by the Cox proportional hazards regression model. Abbreviations: DE-miRNAs: differentially expressed miRNAs; HR: hazard ratio.

Then we screened the DEGs and DE-miRNAs between FLT3 mutation and wild-type AML using sequencing data from TCGA database. A total of 24 DE-miRNAs (6 upregulated and 18 downregulated) and 250 DEGs (54 upregulated and 196 downregulated) were identified (Table S1). According to the results of overall survival analyses, 7 miRNA (miR-1-3p, miR-10a-3p, miR-10a-5p, miR-133a-3p, miR-99b-5p, miR-151a-5p and miR-598-3p) had prognostic significance in AML (Figure 3, Figure S1). However, the change on expression of miR-151a-5p and miR-598-3p (downregulated) did not match their adverse prognostic roles in OS analyses (miR-151a-5p: HR=1.847, 95%CI: 1.271-2.802, P=0.02; miR-598-3p: HR=1.664, 95%CI: 1.128-2.504, P=0.011). Therefore, miR-1-3p, miR-10a-3p, miR-10a-5p, miR-133a-3p and miR-99b-5p were selected as the candidate terms for further researches.

### GO and KEGG enrichment analysis of DEGs

**Figure 4:**
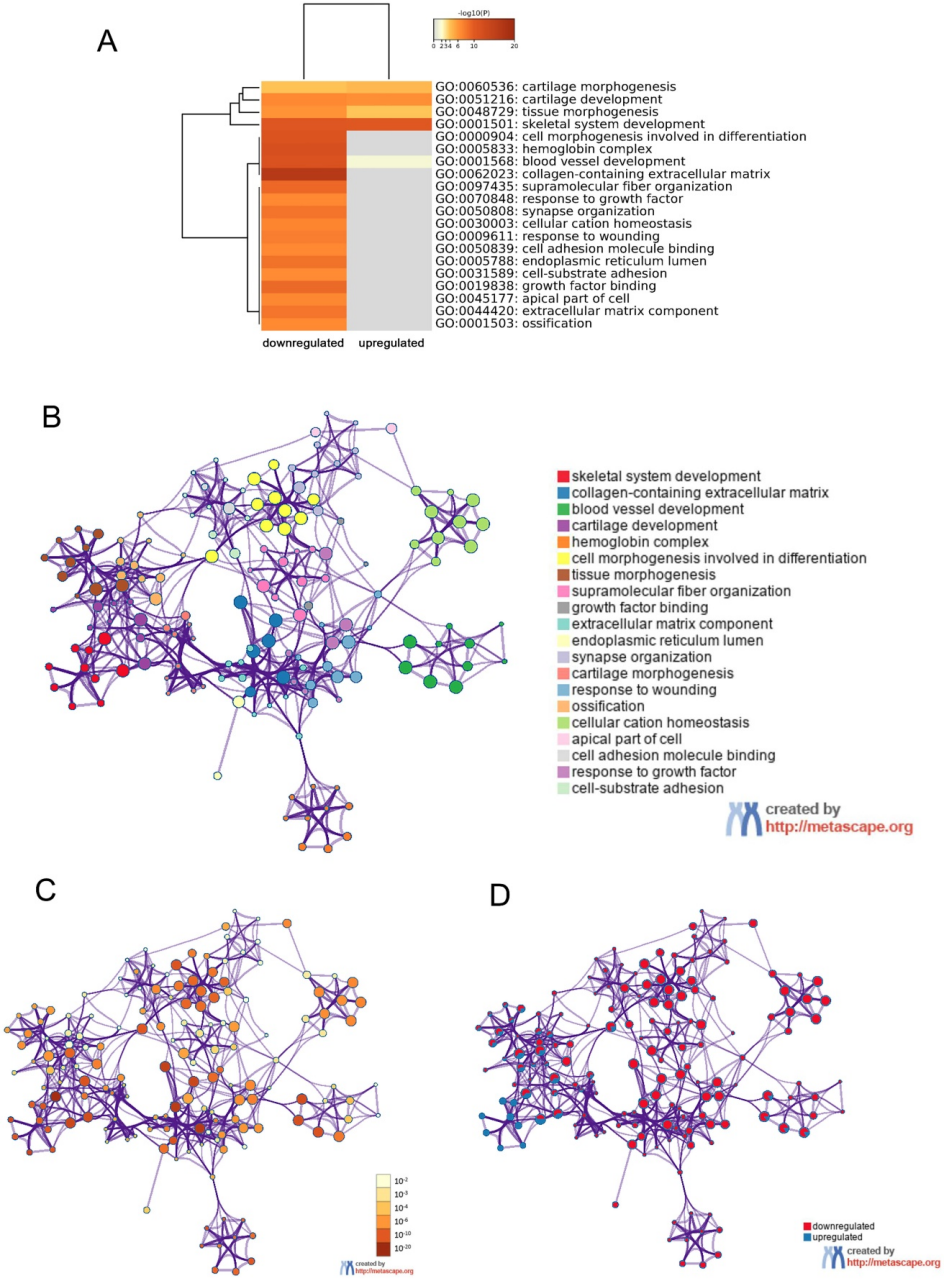
** GO enrichment and interactome analysis of DEGs.** A, Heatmap of enriched terms for downregulated and upregulated DEGs colored by P values was visualized by Metascape. B-D, Network of enriched terms: (B) colored by enriched terms, (C) colored by P value, (D) color-coded based on the identities of the gene lists. Abbreviations: DEGs: differentially expressed genes; GO: gene ontology.

In order to further understand the pathway and process affected by identified DEGs, gene ontology (GO) and Kyoto Encyclopedia of Gene and Genome (KEGG) analyses were respectively performed using Metascape and DAVID. The top 20 clusters with their representative enriched terms of GO analysis were shown in Table 2. The upregulated DEGs were significantly enriched in skeletal system development, blood vessel development, cartilage development, tissue morphogenesis and cartilage morphogenesis. While the downregulated DEGs were mainly enriched in skeletal system development, cell morphogenesis involved in differentiation, tissue morphogenesis, response to growth factor, cell-substrate adhesion and so on. These results were visualized by Metascape. A cluster heat map about enrichment analysis was shown in Figure 4A. Further, enrichment networks have been constructed to show the associations between GO terms. In the networks, the nodes were represented as different terms, which were colored by their cluster ID (Figure 4B), P value (Figure 4C) and the identities of the gene lists (Figure 4D).

**Figure 5:**
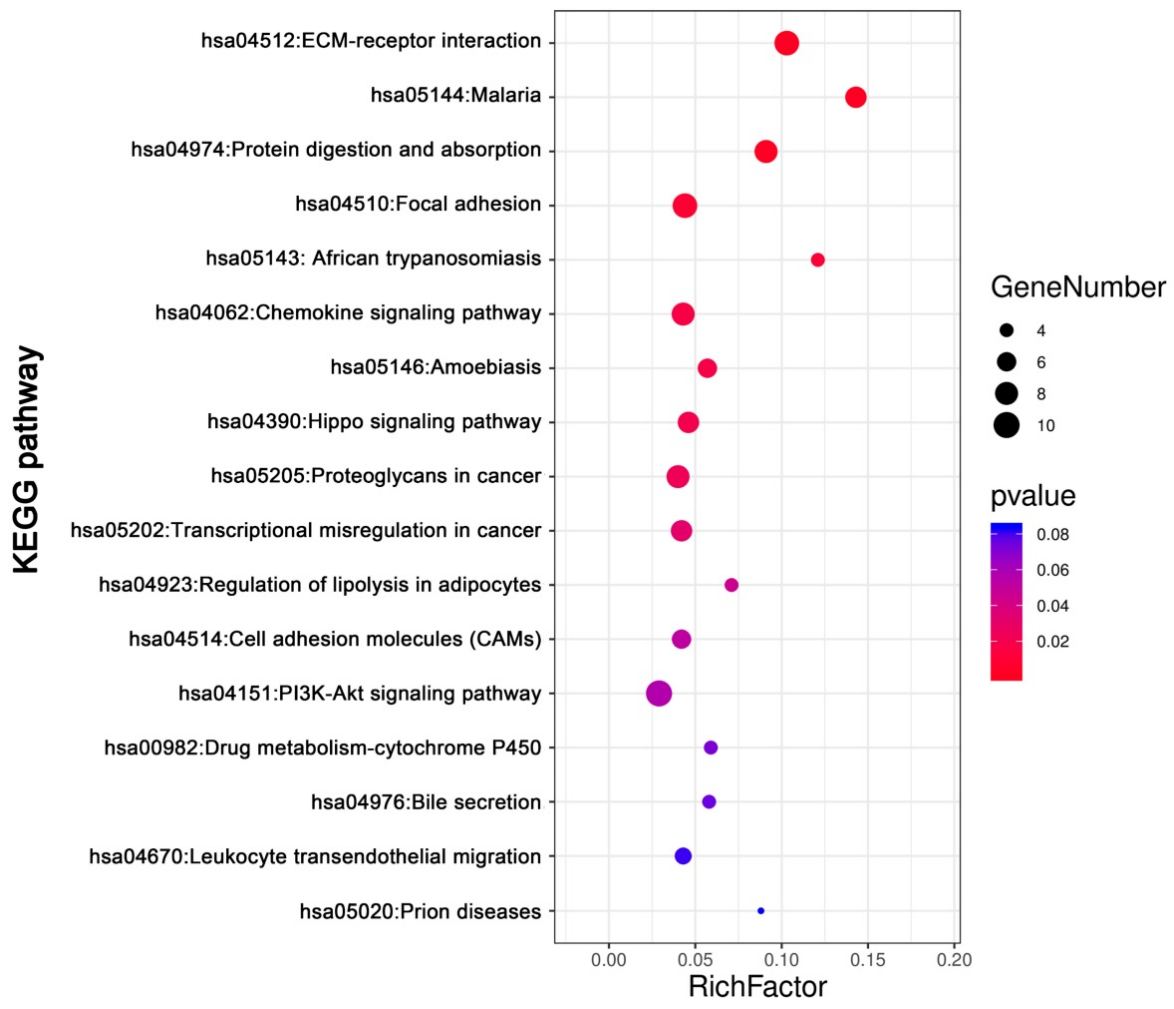
** The KEGG pathways analysis of DEGs.** The KEGG pathways enrichment analysis of differentially expressed genes was perform by DAVID. Abbreviations: KEGG: Kyoto Encyclopedia of Genes and Genomes.

KEGG pathway analysis was conducted for total DEGs by DAVID. The result showed DEGs were obviously enriched in PI3K-Akt signaling pathway, ECM-receptor interaction, focal adhesion Protein digestion and absorption, Chemokine signaling pathway and so on (Figure 5, Table 3).

### PPI network and module analysis of DEGs

**Figure 6:**
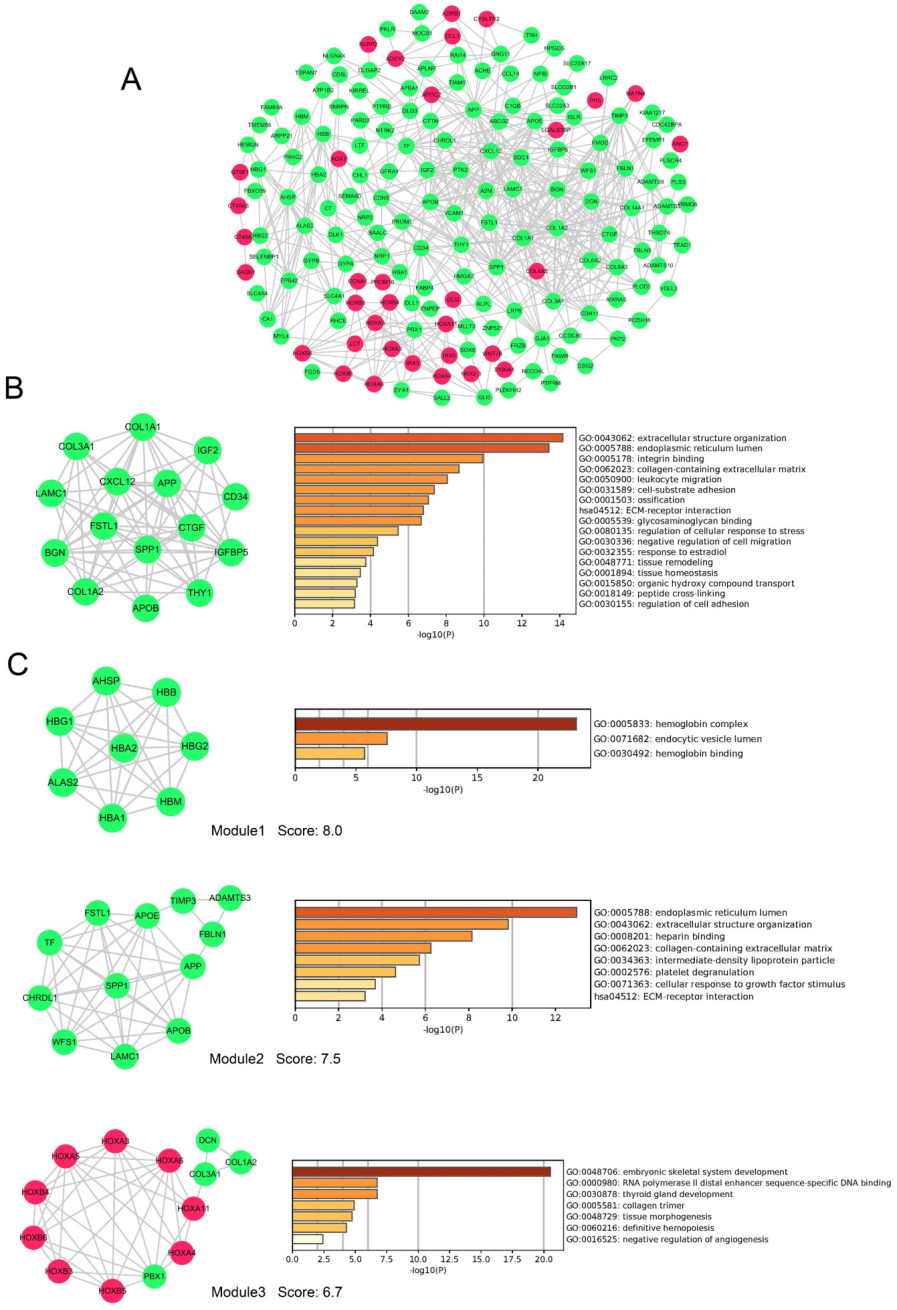
** PPI network, hub genes network and modules analyses of DEGs.** A, PPI network of DEGs. B, The network of 15 hub genes with a higher degree of connectivity and enrichment analysis of these genes. C, Genes of top 3 modules were performed GO and KEGG enrichment analysis by Metascape. Nodes were color-coded based on the expression of DEGs (red, upregulated; green, downregulated).Abbreviations: DEGs: differentially expressed genes; GO: Gene ontology; KEGG: Kyoto Encyclopedia of Genes and Genomes, PPI: protein-protein interaction.

To evaluate the association of DEGs, protein-protein interactome (PPI) network were performed using STRING and Cytoscape software (Figure 6A). The top 20 hub genes were detected by cytoHubba plugin used 12 different algorithms (Table S2). Then, detected by more than five algorithms, 15 hub genes with higher degree of connectivity were selected to build the hub gene PPI network (Figure 6B). The enrichment analysis showed that the cancer-related processes hub genes enrich in were extracellular structure organization, leukocyte migration, cell-substrate adhesion, negative regulation of cell migration, regulation of cell adhesion and so on.

In addition, 9 modules in PPI network were detected by the MCODE plugin. We screened the top 3 significant modules to further analyses. The enrichment analysis demonstrated that genes in modules were mainly enriched in hemoglobin binding, extracellular structure organization, platelet degranulation, ECM-receptor interaction, tissue morphogenesis, and definitive hemopoiesis (Figure 6 C). Seven genes (LAMC1, COL3A1, APOB, COL1A2, APP, SPP1 and FSTL1) were simultaneously identified by hub gene analysis and module analysis.

### miRNA-mRNA regulatory network analysis

**Figure 7:**
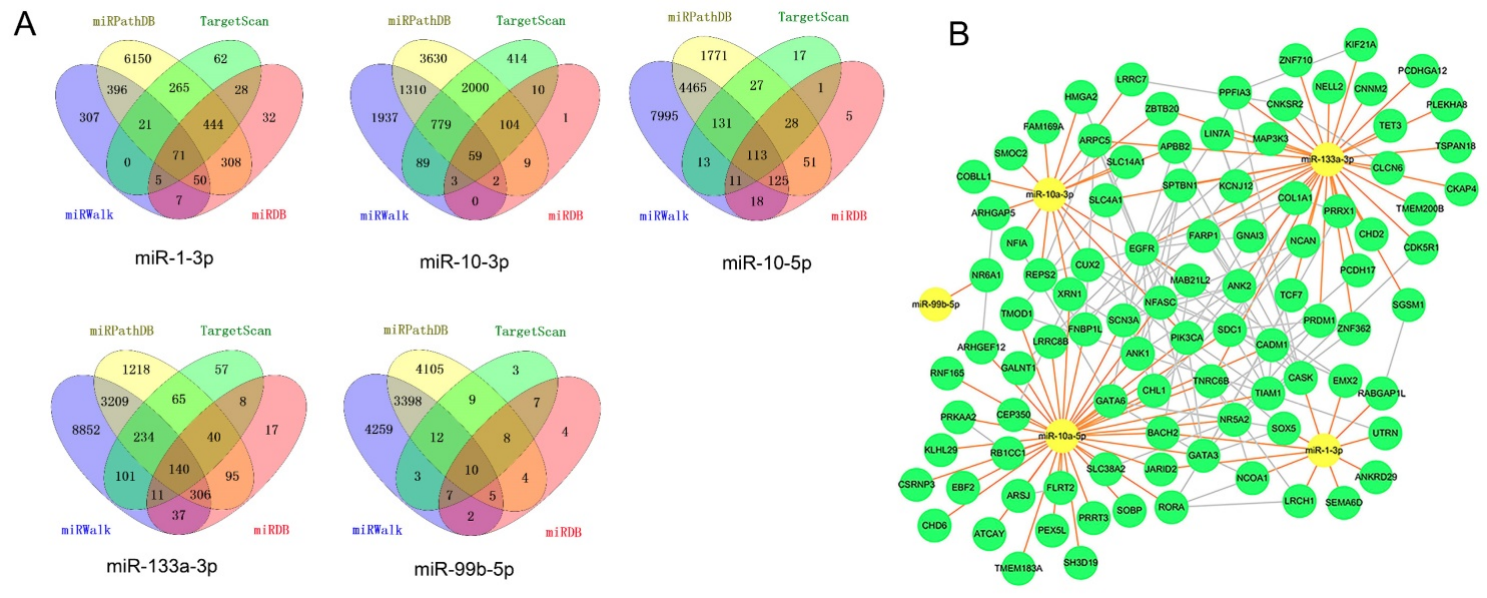
** miRNA-mRNA regulatory network.** A, Diagrams illustrating in prediction of target genes of five candidate miRNAs. B, miRNA-mRNA regulatory network. Nodes were color-coded based on components (yellow, DE-miRNAs; green, target DEGs). The orange lines indicated the regulation relationship between DE-miRNAs and their targets DGEs. The silver lines indicated the relationship between DEGs. Abbreviations: DE-miRNAs: differentially expressed miRNAs: DEGs, differentially expressed genes.

Four target prediction databases were utilized to identify the target genes of five candidate DE-miRNAs (Figure 7A).

The total target genes were matched with DGEs (P<0.05, not limited fold change), while the target genes of upregulated miRNA were corresponded with downregulated genes and target gens of downregulated miRNA were corresponded with upregulated genes. Herein, we totally identified 393 target genes and there were 94 genes expressing differently between FLT3 mutation and wild-type AML. Then, miRNA-mRNA regulatory network was constructed with DE-miRNA (n=5) and target gens (n=94). It showed that miR-1-3p, miR-10a-3p, miR-10a-5p and miR-133a-3p had rich external connections (Figure 7B). Finally, three transcription factors, RORA, CUX2 and ZNF362, were found in DE-miRNAs-DEGs regulatory network using by OmicsBean (http://www.omicsbean.cn).

### Target genes analysis of miR-10a-3p

Some studies have widely investigated the identified key miRNA in cancers except miR-10a-3p. For example, miR-1-3p acted as a tumor suppressor in acute myeloid leukemia 28, bladder cancer 29, non-small-cell lung cancer 30, hepatocellular carcinoma 31 and so on. Zhi Y et al. reported that high miR-10a-5p expression was associated with poorer overall survival of AML patients 32. miR-133a-3p played a regulated role in prostate cancer 33, ovarian cancer 34 and breast cancer 35. And miR-99b-5p expression was related with gastric cancer 36, colorectal cancer 37 and clear cell renal cell carcinoma 38. However, the molecular mechanism of miR-10a-3p in cancer was still unclear. So, we selected miR-10a-3p for further study.

**Figure 8:**
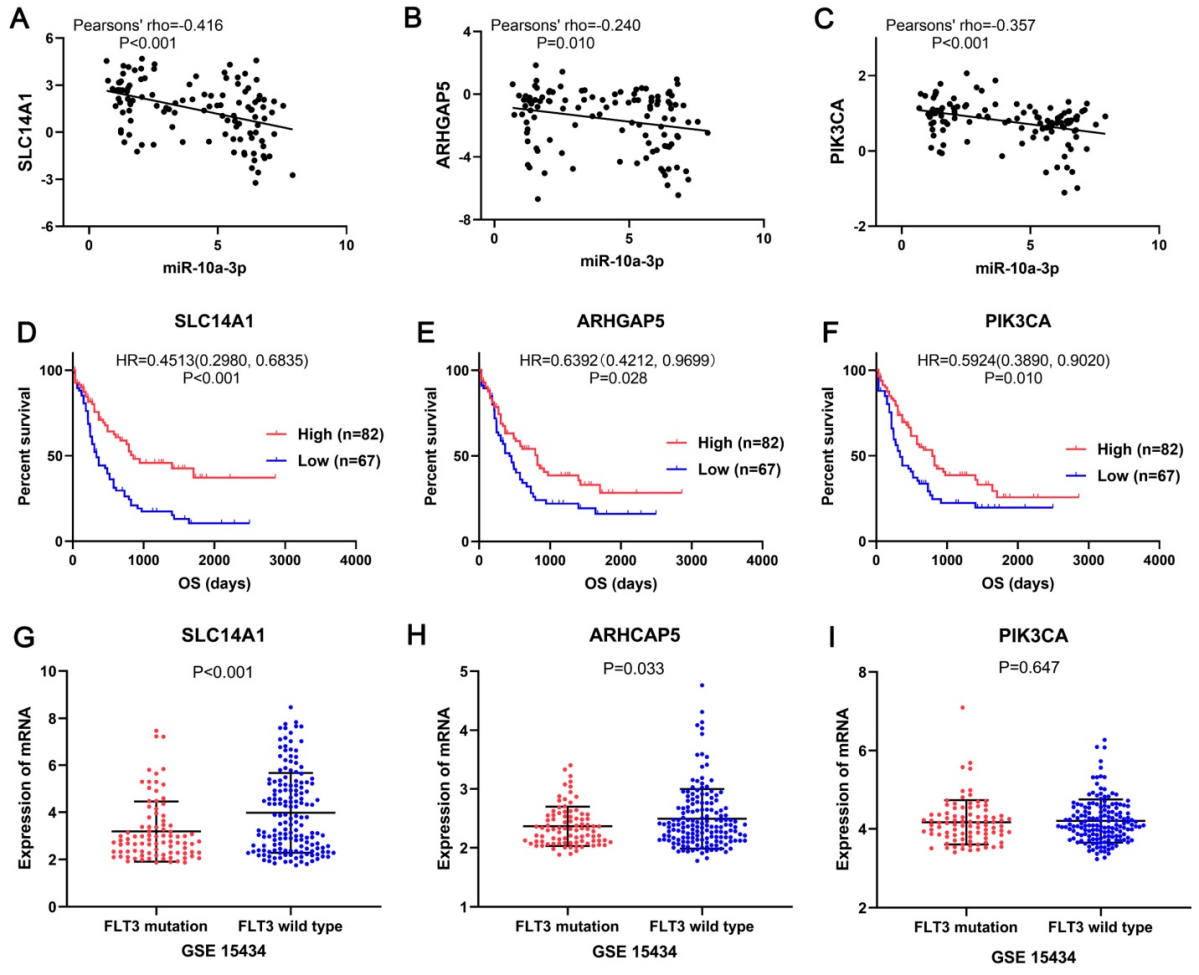
** SLC14A1, ARHGAP5 and PIK3CA may be the target genes of miR-10a-3p involved in AML with FLT3 mutation.** The correlation analyses showed miR-10a-3p had significant negative correlations with SLC14A1 (pearsons' rho=-0.42), ARHGAP5 (pearsons' rho=-0.24) and PIK3CA (pearsons' rho=-0.36) in AML with FLT3 mutation from TCGA. Survival analyses shown that SLC14A1, ARHGAP5 and PIK3CA had better overall survival (OS) of AML patients form TCGA and had different mRNA expression levels between FLT3 mutation and wild-type AML from GSE15434. Abbreviations: TCGA: The Cancer Genome Atlas; OS: overall survival.

In the miRNA-mRNA regulatory network, miR-10a-3p had 15 target genes: HMGA2, NFIA, CUX2, SLC14A1, LRRC7, APBB2, SMOC2, FAM169A, ARHGAP5, COBLL1, MAB21L2, ZBTB20, REPS2, XRN1 and PIK3CA, also acted as downregulated genes in FLT3 mutation AML of TCGA. Then these target genes and miR-10a-3p were performed correlation analysis. The results shown that miR-10a-3p had negative correlations with HMGA2, NFIA, CUX2, SLC14A1, APBB2, ARHGAP5, COBLL1, REPS2, XRN1 and PIK3CA (P<0.05, Figure 8, Figure S2), of which SLC14A1, ARHGAP5, PIK3CA had prognostic significance in OS of AML patients (P<0.05, Figure 8, Figure S2). Simultaneously, expression of these three target genes was verified in GSE15434. Expression of SLC14A1 (P<0.001) and ARHGAP5 (P=0.033) were both reduced in AML with FLT3 mutation, but there was no obvious difference of PIK3CA (P=0.647) expression (Figure 8).

## Discussion

Acute myeloid leukemia (AML) is a common hematological malignancy which the incidence is increasing 1. As a highly heterogeneous disease, AML is associated with many different cytogenetic abnormalities and genetic alterations detected at diagnosis. Hence, the study of valuable biomarkers is beneficial to enhance our understanding of AML pathogenesis. FMS-like tyrosine kinase 3 (FLT3) mutation is one of the most common genetic abnormalities in AML patients 7. Survival analyses indicated that FLT3 mutation was significantly associated with poor OS, RFS and EFS using data provided by Bullinger L et al. 14, which was consisted with previous studies 39, 40. Therefore, further investigation is important for better understanding the biological roles of FLT3 mutations in AML.

In present study, we firstly used mRNA and miRNA sequencing data from TCGA database to analyze the molecular correlates of FLT3 mutations in adult AML. A total of 24 DE-miRNAs (6 up-regulated and 18 down-regulated) and 250 DEGs (54 up-regulated and 196 down-regulated) were identified using bioinformatics analysis. To understand the changes in processes and pathways FLT3 mutation caused, GO terms and KEGG pathway enrichment analyses were performed. The results of GO analyses showed DEGs were notably abundant in skeletal system development, blood vessel development, cartilage development, tissue morphogenesis, cartilage morphogenesis, cell morphogenesis involved in differentiation, response to growth factor, cell-substrate adhesion and so on. It is suggested that FLT3 mutation affected leukemia via these processes.

The KEGG pathway analysis showed that DEGs were obviously enriched in PI3K-Akt signaling pathway, ECM-receptor interaction and focal adhesion. Previous studies had reported that PI3K-Akt signaling pathway was involved in AML 41, 42. But the roles of ECM-receptor interaction pathway and focal adhesion pathway in AML were still unclear. Kim SH et al demonstrated that ECM-receptor interaction led to direct or indirect control of cell adhesion, migration, differentiation, proliferation and apoptosis 43. ECM-receptor interaction was reported to be related to esophageal squamous cell carcinoma 44 and epithelial ovarian cancer 45. In addition, focal adhesion signaling hubs critically regulated cell behavior, impacted on tumor cell survival and served as potential cancer targets 46.

PPI network showed DEGs involved in FLT3 mutation were rich interactions. The top hub genes detected by more than five algorithms included LAMC1, COL3A1, COL1A1, IGF2, CD34, IGFBP5, THY1, APOB, COL1A2, BGN, CXCL12, APP, CTGF, SPP1 and FSTL1. By enrichment analysis, these hub genes were associated with extracellular structure organization, leukocyte migration, cell-substrate adhesion, negative regulation of cell migration, regulation of cell adhesion and so on. In addition, the analyses demonstrated that genes in the top 3 significant modules were mainly enriched in hemoglobin binding, extracellular structure organization, platelet degranulation, ECM-receptor interaction, tissue morphogenesis, and definitive hemopoiesis. Seven genes (LAMC1, COL3A1, APOB, COL1A2, APP, SPP1 and FSTL1) were simultaneously identified by hub gene analysis and module analysis. Therefore, these genes might be crucial in FLT3 mutation AML.

We identified 24 DE-miRNAs and 5 of them had prognostic value and the results matched their expression levels. Four miRNAs (miR-1-3p, miR-10a-3p, miR-10a-5p and miR-133a-3p) resulted in adverse outcome and miR-99b-5p played positive prognosis in AML. Four target prediction databases were used to identify the targets of DE-miRNAs. Then, miRNA-mRNA regulatory network revealed that miR-1-3p, miR-10a-3p, miR-10a-5p and miR-133a-3p had rich external connections, while miR-99b-5p have few external connections. miR-1-3p acted as a tumor suppressor in acute myeloid leukemia 28, bladder cancer 29, non-small-cell lung cancer 30, hepatocellular carcinoma 31 and so on. Zhi Y et al. reported that high miR-10a-5p expression was associated with poorer overall survival of AML patients 32. miR-133a-3p played a regulated role in prostate cancer 33, ovarian cancer 34 and breast cancer 35. And miR-99b-5p expression was related with gastric cancer 36, colorectal cancer 37 and clear cell renal cell carcinoma 38. However, the study about role of miR-10a-3p in cancer was still lacking. So, we selected miR-10a-3p for further study.

Among the target genes of miR-10a-3p, SLC14A1, ARHGAP5, PIK3CA had significant negative correlation with it and led to better overall survival in AML. Simultaneously, expression of these three target genes was verified in GSE15434. Expression of SLC14A1 and ARHGAP5 were both reduced in AML with FLT3 mutation, but there was no obvious difference of PIK3CA expression. Human solute carrier family 14 member 1 (SLC14A1) gene was crucial to the kidney's ability to concentrate urine and also expressed on red blood cells 47. Studies has reported that SLC14A1 was regard as a novel target for human urothelial cancer 48 and urinary bladder cancer 49. However, Rho GTPase activating protein 5 (ARHGAP5) was identified as an oncogene that affected cell migration and invasion in cervical cancer 50, gastric cancer 51 and nasopharyngeal carcinoma 52. Phosphatidylinositol 3-kinase catalytic subunit alpha (PIK3CA) mutation has found in hematological malignancies 53, including acute myeloid leukemia 54 and diffuse large B cell lymphoma 55. In addition, studies of SLC14A1 and ARHGAP5 related to AML had not been reported. According to the results, the expression levels of SLC14A1 and ARHGAP5 were downregulated in FLT3 mutation AML patients compared with the wild-type, which were required to be confirm by further experiment.

## Conclusion

Our study indicated that mutant FLT3 result in poor prognosis in adult AML which was in line with previous reports. We successfully identified molecular markers, processes and pathways affected by FLT3 mutation in AML. Furthermore, miR-10a-3p, a novel biomarker, might involve in the development of FLT3 mutation AML by targeting SLC14A1, ARHGAP5 and PIK3CA. However, further experiments are still required to support our results.

## Supplementary Material

Supplementary figures and tables.

## Figures and Tables

**Table 1:** Basic information of enrolled datasets

Datasets	Type	FLT3 mutation (n)	FLT3 wild-type (n)	Total (n)	Purpose
Bullinger L et al.	survival data	70	106	176	Survival analysis of FLT3 mutation AML
TCGA	miRNA	54	131	185	DE-miRNAs
	mRNA	49	121	170	DEGs
	survival data	-	-	-	Survival analysis of AML with DE-miRNAs and target genes
GSE15434	mRNA	90	161	251	Evaluation of target genes expression

Abbreviations: TCGA: The Cancer Genome Atlas; FLT3: FMS-like tyrosine kinase 3, AML: acute myeloid leukemia; DE-miRNAs: differentially expressed miRNAs; DEGs: differentially expressed genes

**Table 2:** Top 20 GO terms enrichment analysis of DEGs by Metascape

Gene	GO	Category	Description	Count	%	Log10(P)	Log10(q)
**↓↑**	GO:0001501	GO BP	skeletal system development	36	14.63	-19.06	-14.71
**↓**	GO:0062023	GO CC	collagen-containing extracellular matrix	30	12.20	-16.60	-12.55
**↓↑**	GO:0001568	GO BP	blood vessel development	34	13.82	-12.31	-8.82
**↓↑**	GO:0051216	GO BP	cartilage development	19	7.72	-12.27	-8.82
**↓**	GO:0005833	GO CC	hemoglobin complex	7	3.63	-11.85	-8.34
**↓**	GO:0000904	GO BP	cell morphogenesis involved in differentiation	28	14.51	-10.99	-7.72
**↓↑**	GO:0048729	GO BP	tissue morphogenesis	28	11.38	-9.47	-6.41
**↓**	GO:0097435	GO BP	supramolecular fiber organization	24	12.44	-8.99	-6.14
**↓**	GO:0019838	GO MF	growth factor binding	12	6.22	-8.92	-6.10
**↓**	GO:0044420	GO CC	extracellular matrix component	9	3.66	-8.67	-5.74
**↓**	GO:0005788	GO CC	endoplasmic reticulum lumen	18	7.32	-8.48	-5.60
**↓**	GO:0050808	GO BP	synapse organization	18	9.33	-8.26	-5.52
**↓↑**	GO:0060536	GO BP	cartilage morphogenesis	7	2.85	-7.87	-5.16
**↓**	GO:0009611	GO BP	response to wounding	22	11.40	-7.50	-4.89
**↓**	GO:0001503	GO BP	ossification	19	7.72	-7.50	-4.85
**↓**	GO:0030003	GO BP	cellular cation homeostasis	21	10.88	-7.16	-4.58
**↓**	GO:0045177	GO CC	apical part of cell	16	8.29	-7.02	-4.46
**↓**	GO:0050839	GO MF	cell adhesion molecule binding	18	9.33	-6.91	-4.38
**↓**	GO:0070848	GO BP	response to growth factor	22	11.40	-6.89	-4.37
**↓**	GO:0031589	GO BP	cell-substrate adhesion	15	7.77	-6.71	-4.21

Abbreviations: **↓**: downregulated genes; **↑**: upregulated genes; GO: gene ontology; BP: biological processes; CC: cellular components; MF: molecular function; DEGs: differentially expressed genes

**Table 3:** The KEGG pathways enrichment analysis of DEGs by DAVID

ID	Term	Count	%	P value	Genes
hsa04151	PI3K-Akt signaling pathway	10	0.030149	0.056461	PTK2, COL3A1, COL6A3, COL1A2, COL6A2, GNG11, COL1A1, LAMC1, SPP1, COL4A5
hsa04512	ECM-receptor interaction	9	0.027134	2.95E-05	SDC1, COL3A1, COL6A3, COL1A2, COL6A2, COL1A1, LAMC1, SPP1, COL4A5
hsa04510	Focal adhesion	9	0.027134	0.008848	PTK2, COL3A1, COL6A3, COL1A2, COL6A2, COL1A1, LAMC1, SPP1, COL4A5
hsa04974	Protein digestion and absorption	8	0.024119	2.38E-04	COL14A1, ATP1B2, COL3A1, COL6A3, COL1A2, COL6A2, COL1A1, COL4A5
hsa04062	Chemokine signaling pathway	8	0.024119	0.01651	CCL1, PTK2, PARD3, CCL14, ADCY2, TIAM1, GNG11, CXCL12
hsa05205	Proteoglycans in cancer	8	0.024119	0.023575	WNT7B, PTK2, CTTN, SDC1, TIAM1, IGF2, DCN, TIMP3
hsa05144	Malaria	7	0.021104	6.11E-05	GYPB, VCAM1, SDC1, GYPA, HBA2, HBA1, HBB
hsa04390	Hippo signaling pathway	7	0.021104	0.020389	WNT7B, PARD3, FRMD6, CTGF, DLG3, TEAD1, GLI2
hsa05202	Transcriptional misregulation in cancer	7	0.021104	0.031476	PROM1, EYA1, PTK2, TSPAN7, PBX1, HMGA2, MLLT3
hsa05146	Amoebiasis	6	0.018089	0.017315	PTK2, COL3A1, COL1A2, COL1A1, LAMC1, COL4A5
hsa04514	Cell adhesion molecules (CAMs)	6	0.018089	0.05155	VCAM1, SDC1, PTPRM, CD34, NLGN4X, CDH5
hsa04670	Leukocyte transendothelial migration	5	0.015074	0.08114	VCAM1, PTK2, CXCL12, CDH5, THY1
hsa05143	African trypanosomiasis	4	0.012059	0.011226	VCAM1, HBA2, HBA1, HBB
hsa04923	Regulation of lipolysis in adipocytes	4	0.012059	0.045163	ADCY2, ADRB1, FABP4, PRKG2
hsa00982	Drug metabolism - cytochrome P450	4	0.012059	0.072398	FMO2, FMO3, UGT2B11, GSTT1
hsa04976	Bile secretion	4	0.012059	0.074937	ADCY2, ATP1B2, SLC4A4, ABCG2
hsa05020	Prion diseases	3	0.009045	0.083993	C7, C1QB, LAMC1

Abbreviations: KEGG: Kyoto Encyclopedia of Genes and Genomes; DEGs: differentially expressed genes.
