# Supplementary material for: Identification of the key genes and microRNAs in adult acute myeloid leukemia with FLT3 mutation by bioinformatics analysis
Source: Int J Med Sci. 2020 May 18;17(9):1269–80. doi: 10.7150/ijms.46441 (PMC7294926; doi:10.7150/ijms.46441)
Supplement: Supplementary file 1 — Supplementary figures and tables. [file ijmsv17p1269s1.pdf]

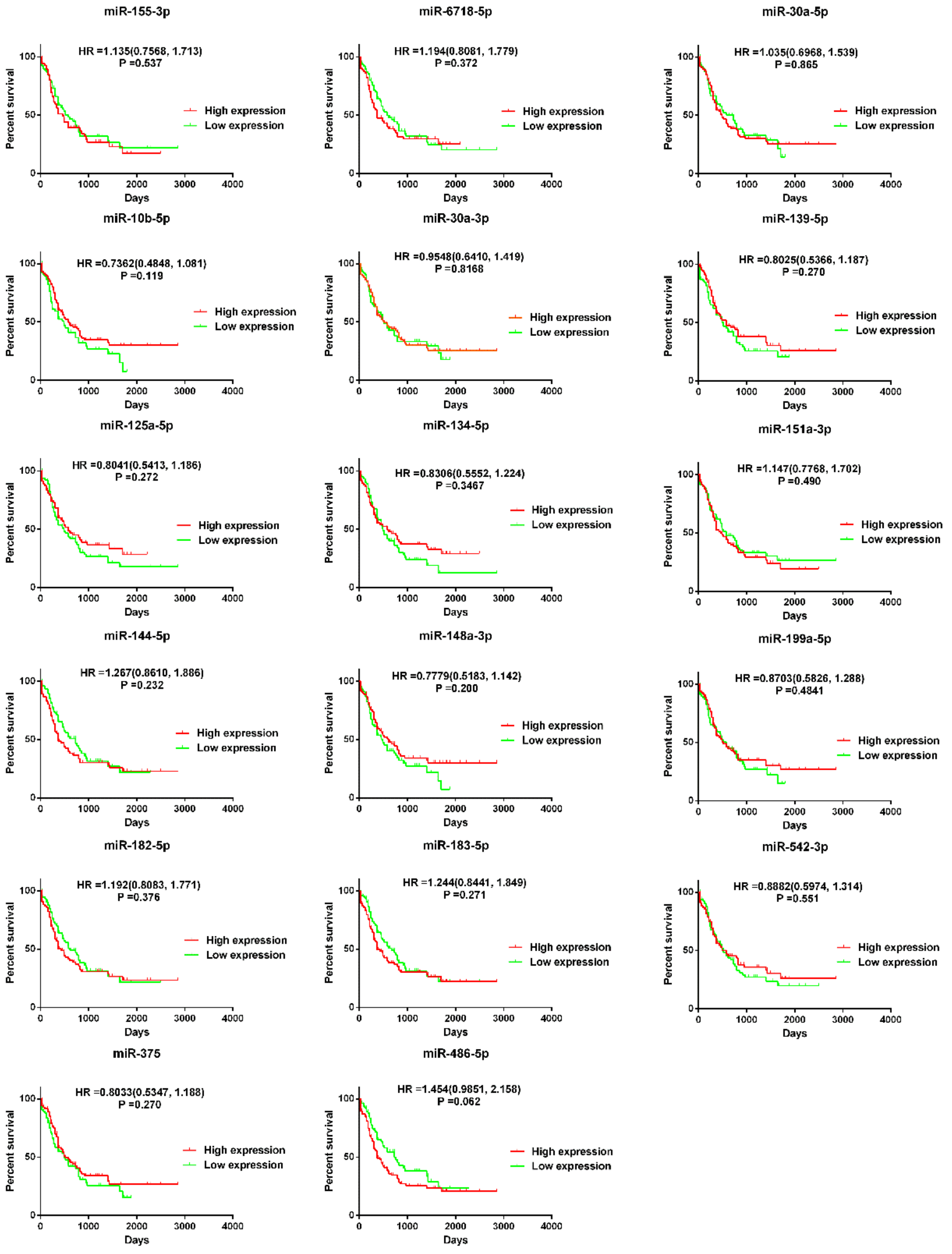

Figure S1 Prognostic significance of seventeen DE-miRNAs

Kaplan-Meier survival curves of overall survival (OS) in AML was performed using TCGA dataset. The top 50% miRNA expression was defined as high expression group and the rest was defined as low expression group. The hazard ratio (HR) with 95% confidence intervals (CIs) were analyzed by the Cox proportional hazards regression model. Seventeen miRNAs had no statistical difference in survival analyses.

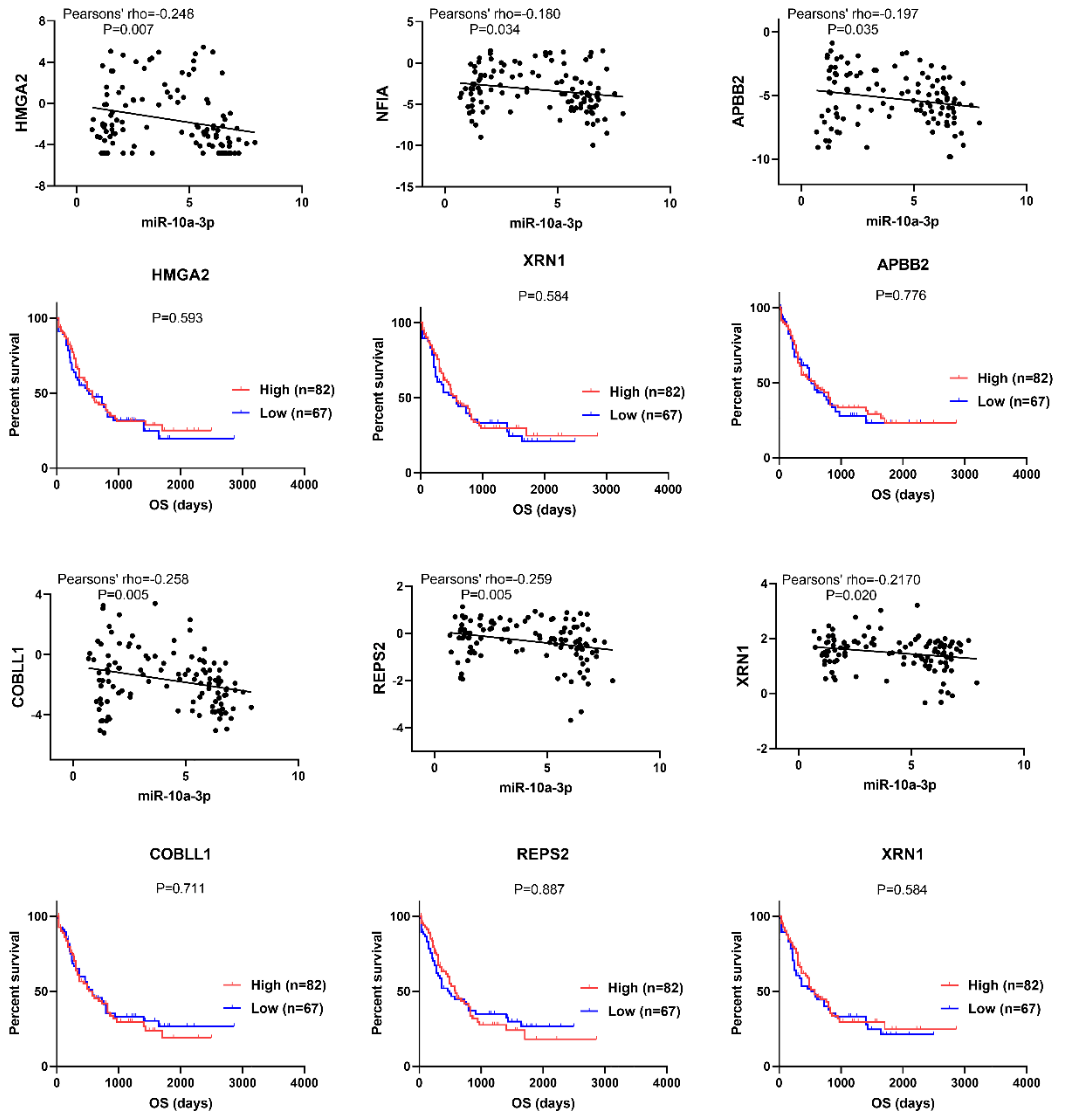

Figure S2 Survival analyses of six target genes negative correlated with miR-10a-3p

Kaplan-Meier survival curves of overall survival (OS) in AML was performed using TCGA dataset.

Table S1 Differentially expressed genes and miRNAs form TCGA dataset

Type	Upregulated	Downregulated
miRNA	miR-1-3p, miR-10a-3p, miR-10a-5p, miR-133a-3p, miR-155-3p, miR-6718-5p (n=6)	miR-148a-3p, miR-199a-5p, miR-10b-5p, miR-182-5p, miR-139-5p, miR-486-5p, miR-30a-3p, miR-30a-5p, miR-183-5p, miR-144-5p, miR-99b-5p, miR-125a-5p, miR-542-3p, miR-598-3p, miR-151a-3p, miR-375, miR-151a-5p, miR-134-5p (n=18)
mRNA	NKX2-3, PRDM16, GTSF1, COL4A5, FLJ42875, HOXB6, C20orf200, FAM47E, CT45A1, GLI2, APOC2, HOXB3, LGALS3BP, AGXT, HOXB5, CCL1, HOXB4, WNT7B, CCNA1, CT45A3, LOC728606, ADRB1, HOXA6, IRX3, C6orf97, CYSLTR2, HOXA5, H2AFY2, JPH1, ADCY2, KRT17, HOXA4, SAGE1, TOM1L1, FAM124A, DAPL1, C10orf140, IGSF10, VENTX, BEND6, SCHIP1, NLRP2, HOXA3, C20orf54, LCT, APOL4, ANO7, HOXA11AS, MACC1, S100A1, IRX5, HOXA11, MATN4, PI15 (n=54)	CXCL12, PXDN, TSPAN7, CHRDL1, FAM171A1, TRH, VCAM1, KIRREL, ZNF521, ADAMTS3, LAMC1, HBA1, COL1A2, FAM64A, DSG2, PKLR, SLCO5A1, COL3A1, MYEF2, CD5L, MXRA5, SNORD116-4, MYCT1, AHSP, HBA2, DCN, HBB, GYPA, DLGAP2, IPW, PBX1, CDC42BPA, C7, APP, FSTL1, TMEM56, ARPP21, ALAS2, SLC4A1, THY1, LTF, PAWR, THSD7A, CYYR1, MCF2L, FGD5, CHL1, GNG11, COL6A3, SVEP1, SNORD116-28, AMPD1, LRP6, GJA1, PROM1, ADAMTS10, APOE, NRP1, PAR-SN, FMO2, HBG2, SEMA6D, LIFR, TEAD1, C10orf58, FRZB, TMIGD2, BGN, RAB3IL1, DNASE1L3, CD34, CCDC80, SNRPN, PTPRD, PCDH18, SDC1, COL1A1, NRP2, ADAMTS9, SLC4A4, CDH11, IGF2, CA1, DLK1, EPB42, PAR5, ZNF626, VGLL3, GPR173, C1QB, ACHE, ALPL, NFATC4, TUSC1, EFEMP1, A2M, SPP1, BAALC, IGFBP5, ATP1B2, FABP4, PARD3, CCL14, SNORD116-20, SDK2, SHD, KIAA1217, C1QTNF1, GLI3, GFRA1, ADD2, TF, C17orf28, LGMN, DLL1, APLNR, HMGA2, FER1L4, LBP, PTPRM, TCTEX1D1, CTGF, ABCG2, APOB, PLEKHH2, GYPB, IKZF2, APBA1, HBG1, PTK2, SELENBP1, PKP2, FBLN5, UGT2B11, PLS3, FMOD, SALL2, NTRK2, TIMP3, ESM1, NFIB, ISLR, LOC96610, SLC22A17, SOX6, HPGDS, FRMD6, MLLT3, ADAM6, SLC22A3, KLF12, CCDC8, GSTT1, NECAB1, FBLN1, TMEM98, PLSCR4, KRT1, ZNF667, COL14A1, ABCA8, NLGN4X, HBM, FAM178B, ZNF704, PLOD2, ENPEP, SEC14L4, CDH5, EYA1, AASS, C1orf21, DAAM2, DLG3, HEMGN, NEDD4L, FLJ22536, RHCE, COL6A2, FMO3, PRKG2, SASH1, TMEM132C, SLCO2B1, WFS1, RAI14, TRAT1, TIAM1, HDGFRP3, MOCS1, LRRC2, TMEM108, PEAR1, MYL4, FBXO39, CTTN (n=196)

Table S2 The top 20 hub genes detected by 12 different algorithms in cytoHubba plugin

Rank	algorithms											
	Betweenness	BottleNeck	Closeness	Clustering Coefficient	Degree	DMNC	EcCentricity	EPC	MCC	MNC	Radiality	Stress
1	CD34	CD34	SPP1	CHRDL1	APP	CHRDL1	AHSP	COL1A2	IGFBP5	COL1A1	CD34	CD34
2	APP	APP	COL1A1	DSG2	COL1A1	WFS1	HBA1	COL1A1	SPP1	SPP1	SPP1	GYPA
3	CXCL12	HBB	CD34	LRRC2	SPP1	HBM	HBB	SPP1	FSTL1	APP	THY1	HEMGN
4	COL1A1	CXCL12	CXCL12	SOX6	COL1A2	COL14A1	GYPA	COL3A1	LAMC1	COL1A2	CXCL12	APP
5	GYPA	COL1A2	APP	IRX5	COL3A1	HOXB5	GYPB	BGN	APP	COL3A1	COL1A1	GTSF1
6	GJA1	HOXB6	THY1	SLC22A3	CXCL12	HBG1	ALAS2	DCN	APOE	BGN	IGF2	THY1
7	ADCY2	IGF2	COL1A2	MATN4	CD34	HBG2	SLC4A1	APP	APOB	CXCL12	APP	CXCL12
8	COL3A1	PBX1	COL3A1	RHCE	BGN	HOXB3	EPB42	CXCL12	TF	DCN	COL1A2	HBB
9	PBX1	ADCY2	BGN	CCDC80	DCN	COL6A2	HBG1	THY1	WFS1	THY1	BGN	COL1A1
10	HEMGN	SPP1	IGF2	HOXA4	THY1	TF	HOXB6	IGFBP5	CHRDL1	CTGF	FSTL1	ALAS2
11	IGF2	COL3A1	CTGF	MXRA5	CTGF	HOXB6	HBG2	CD34	COL1A2	IGFBP5	CTGF	SPP1
12	SPP1	ALAS2	FSTL1	SNRPN	IGFBP5	FBLN5	HBA2	CTGF	COL3A1	APOE	GJA1	CT45A5
13	THY1	CTGF	APOB	FRZB	APOB	COL6A3	APOB	FSTL1	COL1A1	CD34	CDH5	AHSP
14	HOXA11	APOB	IGFBP5	HBM	FSTL1	HOXA4	LCT	LAMC1	BGN	FSTL1	VCAM1	APOB
15	APOB	PROM1	GJA1	SELENBP1	APOE	MYL4	RHCE	FBLN1	FMOD	FMOD	APOB	EPB42
16	HBB	HOXB4	VCAM1	PKLR	FBLN1	SELENBP1	HEMGN	FMOD	DCN	LAMC1	IGFBP5	GJA1
17	ATP1B2	PTK2	DCN	CYSLTR2	IGF2	CCL1	MYL4	COL6A2	FBLN1	FBLN1	PROM1	COL3A1
18	APBA1	HBA1	LAMC1	THSD7A	FMOD	HOXA6	HBM	APOE	TIMP3	IGF2	COL3A1	IGF2
19	GTSF1	HEMGN	SDC1	ADAMTS10	TIMP3	PLOD2	C7	VCAM1	CTGF	APOB	SDC1	ADCY2
20	HOXB6	GJA1	CDH5	TRH	LAMC1	TIMP3	SELENBP1	APOB	COL6A2	TIMP3	LAMC1	CDH5
